# Supplementary material for: Predictive Factors of Ventilatory Support in Chest Trauma
Source: Life (Basel). 2021 Oct 29;11(11):1154. doi: 10.3390/life11111154 (PMC8621668; doi:10.3390/life11111154)
Supplement: Supplementary file 1 [file life-11-01154-s001.zip › Computer code and software.pdf]

Statistical software: IBM Corp., released 2020, IBM SPSS Statistics for Windows, version 27.0; Armonk, NY, IBM Corp.

Encoding: UTF-8.

```
CTABLES
  /VLABELS VARIABLES=Age Age≥65 Age≥75 ASA RTSprehospitalization SpO2prehospitalization
    GCSprehospitalization RTSinED SpO2inED GCSinED SystolicbloodpressureinED TraumaMechanisms
    Ethanolemiatestpositive BE Lactatevalues EFAST ChestXRay TorsoCT Decompressiveminithoracotomy
    Rightchesttubethoracostomy Leftchesttubethoracostomy Emergencysurgery n°ribfractures
Ribfractures≤2
    Ribfractures35 Ribfractures≥6 @1standor2ndribfractures Bilateralribfractures
    Heartlargevesselsinjuries Pneumothorax Hemothorax Hemopneumothorax Pulmonarycontusion
    Scapulaclaviclesternum Pleuraleffusion Isolatedchesttrauma Abdomentrauma Extremitytrauma
Headtrauma
    ChestAIS98 AbdomeAIS98 HeadAIS98 ExtremityAIS98 ISS DEATHPROBALITYTRISS98 Intubation NIMV
Outcome
    DISPLAY=LABEL
  /TABLE Age [MEAN, STDDEV, PTILE 25, MEDIAN, PTILE 75] + Age≥65 [COUNT F40.0, COLPCT.COUNT
    PCT40.1] + Age≥75 [COUNT F40.0, COLPCT.COUNT PCT40.1] + ASA [COUNT F40.0, COLPCT.COUNT
PCT40.1] +
    RTSprehospitalization [MEAN, STDDEV, PTILE 25, MEDIAN, PTILE 75] + SpO2prehospitalization [MEAN,
    STDDEV, PTILE 25, MEDIAN, PTILE 75] + GCSprehospitalization [MEAN, STDDEV, PTILE 25, MEDIAN, PTILE
    75] + RTSinED [MEAN, STDDEV, PTILE 25, MEDIAN, PTILE 75] + SpO2inED [MEAN, STDDEV, PTILE 25,
    MEDIAN, PTILE 75] + GCSinED [MEAN, STDDEV, PTILE 25, MEDIAN, PTILE 75] + SystolicbloodpressureinED
    [MEAN, STDDEV, PTILE 25, MEDIAN, PTILE 75] + TraumaMechanisms [COUNT F40.0, COLPCT.COUNT
PCT40.1] +
    Ethanolemiatestpositive [COUNT F40.0, COLPCT.COUNT PCT40.1] + BE [MEAN, STDDEV, PTILE 25,
MEDIAN,
    PTILE 75] + Lactatevalues [MEAN, STDDEV, PTILE 25, MEDIAN, PTILE 75] + EFAST [COUNT F40.0,
    COLPCT.COUNT PCT40.1] + ChestXRay [COUNT F40.0, COLPCT.COUNT PCT40.1] + TorsoCT [COUNT F40.0,
    COLPCT.COUNT PCT40.1] + Decompressiveminithoracotomy [COUNT F40.0, COLPCT.COUNT PCT40.1] +
    Rightchesttubethoracostomy [COUNT F40.0, COLPCT.COUNT PCT40.1] + Leftchesttubethoracostomy
[COUNT
    F40.0, COLPCT.COUNT PCT40.1] + Emergencysurgery [COUNT F40.0, COLPCT.COUNT PCT40.1] +
    n°ribfractures [MEAN, STDDEV, PTILE 25, MEDIAN, PTILE 75] + Ribfractures≤2 [COUNT F40.0,
```

```
   COLPCT.COUNT PCT40.1] + Ribfractures35 [COUNT F40.0, COLPCT.COUNT PCT40.1] + Ribfractures≥6 [COUNT
   F40.0, COLPCT.COUNT PCT40.1] + @1standor2ndribfractures [COUNT F40.0, COLPCT.COUNT PCT40.1] +
   Bilateralribfractures [COUNT F40.0, COLPCT.COUNT PCT40.1] + Heartlargevesselsinjuries [COUNT F40.0,
   COLPCT.COUNT PCT40.1] + Pneumothorax [COUNT F40.0, COLPCT.COUNT PCT40.1] + Hemothorax [COUNT F40.0,
   COLPCT.COUNT PCT40.1] + Hemopneumothorax [COUNT F40.0, COLPCT.COUNT PCT40.1] + Pulmonarycontusion
   [COUNT F40.0, COLPCT.COUNT PCT40.1] + Scapulaclaviclesternum [COUNT F40.0, COLPCT.COUNT PCT40.1] +
   Pleuraleffusion [COUNT F40.0, COLPCT.COUNT PCT40.1] + Isolatedchesttrauma [COUNT F40.0,
   COLPCT.COUNT PCT40.1] + Abdomentrauma [COUNT F40.0, COLPCT.COUNT PCT40.1] + Extremitytrauma [COUNT
   F40.0, COLPCT.COUNT PCT40.1] + Headtrauma [COUNT F40.0, COLPCT.COUNT PCT40.1] + ChestAIS98 [MEAN,
   STDDEV, PTILE 25, MEDIAN, PTILE 75] + AbdomeAIS98 [MEAN, STDDEV, PTILE 25, MEDIAN, PTILE 75] +
   HeadAIS98 [MEAN, STDDEV, PTILE 25, MEDIAN, PTILE 75] + ExtremityAIS98 [MEAN, STDDEV, PTILE 25,
   MEDIAN, PTILE 75] + ISS [MEAN, STDDEV, PTILE 25, MEDIAN, PTILE 75] + DEATHPROBALITYTRISS98 [MEAN,
   STDDEV, PTILE 25, MEDIAN, PTILE 75] BY Intubation + NIMV + Outcome
  /CATEGORIES VARIABLES=Age≥65 Age≥75 ASA TraumaMechanisms Ethanolemiatestpositive EFAST ChestXRay
   TorsoCT Decompressiveminithoracotomy Rightchesttubethoracostomy Leftchesttubethoracostomy
   Emergencysurgery Ribfractures≤2 Ribfractures35 Ribfractures≥6 @1standor2ndribfractures
   Bilateralribfractures Heartlargevesselsinjuries Pneumothorax Hemothorax Hemopneumothorax
   Pulmonarycontusion Scapulaclaviclesternum Pleuraleffusion Isolatedchesttrauma Abdomentrauma
   Extremitytrauma Headtrauma Intubation NIMV Outcome ORDER=A KEY=VALUE EMPTY=EXCLUDE
  /CRITERIA CILEVEL=95.
* Encoding: UTF-8.

CROSSTABS
  /TABLES=Gender Age≥65 Age≥75 ASA TraumaMechanisms Ethanolemiatestpositive EFAST ChestXRay TorsoCT
   Decompressiveminithoracotomy Rightchesttubethoracostomy Leftchesttubethoracostomy Emergencysurgery
```

```
    Ribfractures≤2 Ribfractures35 Ribfractures≥6 @1standor2ndribfractures Bilateralribfractures
    Heartlargevesselsinjuries Pneumothorax Hemothorax Hemopneumothorax Pulmonarycontusion
    Scapulaclaviclesternum Pleuraleffusion Isolatedchesttrauma Abdomentrauma Extremitytrauma
Headtrauma
    BY Intubation
  /FORMAT=AVALUE TABLES
  /STATISTICS=CHISQ
  /CELLS=COUNT
  /COUNT ROUND CELL.


CROSSTABS
  /TABLES=Gender Age≥65 Age≥75 ASA TraumaMechanisms Ethanolemiatestpositive EFAST ChestXRay
TorsoCT
    Decompressiveminithoracotomy Rightchesttubethoracostomy Leftchesttubethoracostomy
Emergencysurgery
    Ribfractures≤2 Ribfractures35 Ribfractures≥6 @1standor2ndribfractures Bilateralribfractures
    Heartlargevesselsinjuries Pneumothorax Hemothorax Hemopneumothorax Pulmonarycontusion
    Scapulaclaviclesternum Pleuraleffusion Isolatedchesttrauma Abdomentrauma Extremitytrauma
Headtrauma
    Intubation BY NIMV
  /FORMAT=AVALUE TABLES
  /STATISTICS=CHISQ
  /CELLS=COUNT
  /COUNT ROUND CELL.


CROSSTABS
  /TABLES=Gender Age≥65 Age≥75 ASA TraumaMechanisms Ethanolemiatestpositive EFAST ChestXRay
TorsoCT
    Decompressiveminithoracotomy Rightchesttubethoracostomy Leftchesttubethoracostomy
Emergencysurgery
```

```
    Ribfractures≤2 Ribfractures35 Ribfractures≥6 @1standor2ndribfractures Bilateralribfractures
    Heartlargevesselsinjuries Pneumothorax Hemothorax Hemopneumothorax Pulmonarycontusion
    Scapulaclaviclesternum Pleuraleffusion Isolatedchesttrauma Abdomentrauma Extremitytrauma
Headtrauma
    Intubation BY Outcome
  /FORMAT=AVALUE TABLES
  /STATISTICS=CHISQ
  /CELLS=COUNT
  /COUNT ROUND CELL.


*Nonparametric Tests: Independent Samples.
NPTESTS
  /INDEPENDENT TEST (Age RTSprehospitalization SpO2prehospitalization GCSprehospitalization RTSinED
    SpO2inED GCSinED SystolicbloodpressureinED BE Lactatevalues n°ribfractures ChestAIS98 AbdomeAIS98
    HeadAIS98 ExtremityAIS98 ISS DEATHPROBALITYTRISS98) GROUP (Intubation) MANN_WHITNEY
    MEDIAN(TESTVALUE=SAMPLE COMPARE=PAIRWISE)
  /MISSING SCOPE=ANALYSIS USERMISSING=EXCLUDE
  /CRITERIA ALPHA=0.05  CILEVEL=95.

*Nonparametric Tests: Independent Samples.
NPTESTS
  /INDEPENDENT TEST (Age RTSprehospitalization SpO2prehospitalization GCSprehospitalization RTSinED
    SpO2inED GCSinED SystolicbloodpressureinED BE Lactatevalues n°ribfractures ChestAIS98 AbdomeAIS98
    HeadAIS98 ExtremityAIS98 ISS DEATHPROBALITYTRISS98) GROUP (NIMV) MANN_WHITNEY
    MEDIAN(TESTVALUE=SAMPLE COMPARE=PAIRWISE)
  /MISSING SCOPE=ANALYSIS USERMISSING=EXCLUDE
  /CRITERIA ALPHA=0.05  CILEVEL=95.

*Nonparametric Tests: Independent Samples.
NPTESTS
  /INDEPENDENT TEST (Age RTSprehospitalization SpO2prehospitalization GCSprehospitalization RTSinED
```

```
   SpO2inED GCSinED SystolicbloodpressureinED BE Lactatevalues n°ribfractures ChestAIS98 AbdomeAIS98
   HeadAIS98 ExtremityAIS98 ISS DEATHPROBALITYTRISS98) GROUP (Outcome) MANN_WHITNEY
  /MISSING SCOPE=ANALYSIS USERMISSING=EXCLUDE
  /CRITERIA ALPHA=0.05  CILEVEL=95.


LOGISTIC REGRESSION VARIABLES Intubation
  /METHOD=ENTER SpO2prehospitalization RTSprehospitalization RTSinED SpO2inED BE Lactatevalues
    Decompressiveminithoracotomy n°ribfractures @1standor2ndribfractures Bilateralribfractures
    Heartlargevesselsinjuries Pneumothorax Hemothorax Hemopneumothorax Pulmonarycontusion
    Scapulaclaviclesternum Pleuraleffusion Isolatedchesttrauma Abdomentrauma Headtrauma
Extremitytrauma
    ISS
  /CONTRAST (Decompressiveminithoracotomy)=Indicator(1)
  /CONTRAST (@1standor2ndribfractures)=Indicator(1)
  /CONTRAST (Bilateralribfractures)=Indicator(1)
  /CONTRAST (Heartlargevesselsinjuries)=Indicator(1)
  /CONTRAST (Pneumothorax)=Indicator(1)
  /CONTRAST (Hemothorax)=Indicator(1)
  /CONTRAST (Hemopneumothorax)=Indicator(1)
  /CONTRAST (Pulmonarycontusion)=Indicator(1)
  /CONTRAST (Scapulaclaviclesternum)=Indicator(1)
  /CONTRAST (Pleuraleffusion)=Indicator(1)
  /CONTRAST (Isolatedchesttrauma)=Indicator(1)
  /CONTRAST (Abdomentrauma)=Indicator(1)
  /CONTRAST (Extremitytrauma)=Indicator(1)
  /CONTRAST (Headtrauma)=Indicator(1)
  /CRITERIA=PIN(.05) POUT(.10) ITERATE(20) CUT(.5).

LOGISTIC REGRESSION VARIABLES Intubation
  /METHOD=ENTER n°ribfractures @1standor2ndribfractures Bilateralribfractures
```

```
  Heartlargevesselsinjuries Pneumothorax Hemothorax Hemopneumothorax Pulmonarycontusion
  Scapulaclaviclesternum Pleuraleffusion Isolatedchesttrauma Abdomentrauma Headtrauma
Extremitytrauma
 /CONTRAST (@1standor2ndribfractures)=Indicator(1)
 /CONTRAST (Bilateralribfractures)=Indicator(1)
 /CONTRAST (Heartlargevesselsinjuries)=Indicator(1)
 /CONTRAST (Pneumothorax)=Indicator(1)
 /CONTRAST (Hemothorax)=Indicator(1)
 /CONTRAST (Hemopneumothorax)=Indicator(1)
 /CONTRAST (Pulmonarycontusion)=Indicator(1)
 /CONTRAST (Scapulaclaviclesternum)=Indicator(1)
 /CONTRAST (Pleuraleffusion)=Indicator(1)
 /CONTRAST (Isolatedchesttrauma)=Indicator(1)
 /CONTRAST (Abdomentrauma)=Indicator(1)
 /CONTRAST (Extremitytrauma)=Indicator(1)
 /CONTRAST (Headtrauma)=Indicator(1)
 /CRITERIA=PIN(.05) POUT(.10) ITERATE(20) CUT(.5).

LOGISTIC REGRESSION VARIABLES Intubation
 /METHOD=ENTER SpO2prehospitalization GCSprehospitalization SpO2inED GCSinED
  SystolicbloodpressureinED BE Lactatevalues
 /CRITERIA=PIN(.05) POUT(.10) ITERATE(20) CUT(.5).

LOGISTIC REGRESSION VARIABLES NIMV
 /METHOD=ENTER SpO2prehospitalization RTSprehospitalization RTSinED Lactatevalues BE
  Decompressiveminithoracotomy Rightchesttubethoracostomy Leftchesttubethoracostomy n°ribfractures
  @1standor2ndribfractures Bilateralribfractures Pneumothorax Hemothorax Pulmonarycontusion
  Scapulaclaviclesternum Pleuraleffusion Isolatedchesttrauma Abdomentrauma Extremitytrauma ISS
  Intubation
 /CONTRAST (@1standor2ndribfractures)=Indicator(1)
 /CONTRAST (Bilateralribfractures)=Indicator(1)
 /CONTRAST (Pneumothorax)=Indicator(1)
```

```
 /CONTRAST (Hemothorax)=Indicator(1)
 /CONTRAST (Pulmonarycontusion)=Indicator(1)
 /CONTRAST (Scapulaclaviclesternum)=Indicator(1)
 /CONTRAST (Pleuraleffusion)=Indicator(1)
 /CONTRAST (Isolatedchesttrauma)=Indicator(1)
 /CONTRAST (Abdomentrauma)=Indicator(1)
 /CONTRAST (Extremitytrauma)=Indicator(1)
 /CONTRAST (Decompressiveminithoracotomy)=Indicator(1)
 /CONTRAST (Rightchesttubethoracostomy)=Indicator(1)
 /CONTRAST (Leftchesttubethoracostomy)=Indicator(1)
 /CONTRAST (Intubation)=Indicator(1)
 /CRITERIA=PIN(.05) POUT(.10) ITERATE(20) CUT(.5).

LOGISTIC REGRESSION VARIABLES Outcome
 /METHOD=ENTER Age≥65 ASA n°ribfractures Bilateralribfractures Hemothorax Pulmonarycontusion
   Pleuraleffusion Isolatedchesttrauma Abdomentrauma Headtrauma
 /CONTRAST (Bilateralribfractures)=Indicator(1)
 /CONTRAST (Hemothorax)=Indicator(1)
 /CONTRAST (Pulmonarycontusion)=Indicator(1)
 /CONTRAST (Pleuraleffusion)=Indicator(1)
 /CONTRAST (Isolatedchesttrauma)=Indicator(1)
 /CONTRAST (Abdomentrauma)=Indicator(1)
 /CONTRAST (Age≥65)=Indicator(1)
 /CONTRAST (ASA)=Indicator(1)
 /CONTRAST (Headtrauma)=Indicator(1)
 /CRITERIA=PIN(.05) POUT(.10) ITERATE(20) CUT(.5).
```
