# Supplementary material for: Predictive Factors of Ventilatory Support in Chest Trauma
Source: Life (Basel). 2021 Oct 29;11(11):1154. doi: 10.3390/life11111154 (PMC8621668; doi:10.3390/life11111154)
Supplement: Supplementary file 1 [file life-11-01154-s001.zip › Table S1 population data.pdf]

Table S1: population data

		POPULATION						
		n	%	Mean	SD	IQR1	Median	IQR3
Gender	female	179	21.51%					
	male	653	78.49%					
Age				51	17	38	49	62
Age >65	yes	651	78.10%					
	no	181	21.70%					
Age >75	yes	737	88.40%					
	no	94	11.30%					
ASA score	1	508	60.90%					
	2	266	31.90%					
	3	55	6.60%					
	4	2	0.20%					
RTS pre-hosp				11	2.256	11	12	12
SpO2 pre-hosp				95	13.533	95	98	99
GCS pre-hosp				13		13	15	15
RTS in ED				10	3.823	4	12	12
SpO2 in ED				97	6.216	96	99	100
GCS in ED				11	5.392	3	15	15
Systolic blood pressure in ED				126	29882	110	130	145
Trauma mechanisms	other	11	1.30%					
	stab wound	1	0.10%					
	gunshot wound	1	0.10%					
	car accident	161	19.30%					
	bike accident	57	6.80%					
	motorcycle accident	256	30.70%					
	pedestrian accident	121	14.50%					
	precipitation	189	22.70%					
	crusching	6	0.70%					
Alcholemia test postive	no	47	5.60%					
	yes	102	12.20%					
BE				-2.733	5.177	-5.25	-2.4	0.4
Lactate values				3.152	2.551	1.64	2.4	3.5
E-FAST	no	622	74.60%					
	yes	188	22.50%					
Chest X-ray	no	141	16.90%					
	yes	691	82.90%					
Torso CT	no	100	12.00%					
	yes	732	87.80%					
Decompressive mini-thoracotomy	no	804	94.40%					
	yes	28	3.40%					
Right chest tube thoracostomy	no	717	86.00%					
	yes	115	13.80%					
Left chest tube thoracostomy	no	714	85.60%					
	yes	118	14.10%					
Emergency surgery	no	520	62.40%					
	yes	312	37.40%					
n° rib fractures				4.77	3.407	2	4	7
Ribs fractures ≤2	no	576	69.10%					
	yes	256	30.70%					
Rib fractures 3-5	no	536	64.30%					
	yes	296	35.50%					
Rib fractures ≥6	no	547	65.50%					
	yes	280	33.60%					
1st and/or 2nd rib fractures	no	509	61.00%					
	yes	322	38.60%					
Bilateral rib fractures	no	652	78.20%					
	yes	180	21.60%					
Heart/large vessels injuries	no	813	97.50%					
	yes	19	2.30%					
Pneumothorax	no	506	60.70%					
	yes	326	39.10%					
Hemothorax	no	790	94.70%					
	yes	42	5.00%					
Hemopneumothorax	no	748	89.70%					
	yes	84	10.10%					
Pulmonary contusion	no	438	52.50%					
	yes	394	47.20%					
Scapula/clavicle/sternum fractures	no	698	83.70%					
	yes	134	16.10%					
Pleural effusion	no	689	82.60%					
	yes	142	17.00%					
Isolated chest trauma	no	666	70.90%					
	yes	166	19.90%					
Abdomen trauma	no	446	53.50%					
	yes	386	46.30%					
Head trauma	no	306	36.70%					
	yes	526	63.10%					
Extremities trauma	no	260	31.20%					
	yes	572	68.60%					
Chest AIS				3	1	3	3	4
Abdomen AIS				3	1	2	2	3
Head AIS				3	1	2	3	4
Extremities AIS				3	1	2	2	4
ISS				26	14	14	24	36
Death Probability				17.415	26.751	1.575	4.8	19
Outcome	survived	755	90.80%					
	deceased	77	9.20%					
Invasive mechanical ventilation	no	596	59.70%					
	yes	336	40.30%					
Non invasive mechanical ventilation	no	569	68.30%					
	yes	181	21.70%					
